# A Mental Health Hospitalization at Home program as a novel healthcare delivery model on the postpartum period

**DOI:** 10.1192/j.eurpsy.2025.778

**Published:** 2025-08-26

**Authors:** O. Marco Estrada, I. Agasi, N. Cabrera, I. Pacchiarotti, I. Pereta, D. N. Ocejo, E. Solé, A. Roca, L. Navarro, M. Garriga

**Affiliations:** 1 Psychiatry; 2 Hospital Clínic de Barcelona, Barcelona, Spain

## Abstract

**Introduction:**

The early postnatal period is at high risk for new and recurrent episodes of severe mental illness, with around one to two women in 1,000 requiring admission in the first few months after birth.

Home visits by midwives/obstetricians/paediatricians have been tested on preventing mental health problems on the postpartum with no home specific treatment when mental ill relapse appears. Indeed, scarce literature is found on acute relapses on mental health on the postpartum in terms of home visiting programs.

**Objectives:**

Authors aim to explore the role of a Mental Health Hospitalization at Home (MH-HaH) program on acute mental health status on the postpartum period.

**Methods:**

A descriptive study on women attended in a HaH-MH program due to an acute mental health crisis on the postpartum period has been conducted.

**Results:**

Ten mother-baby dyad were attended: 7 were on an avoidance admission regimen (two directly referred from the obstetric ward) and 3 were early discharged from a psychiatric inpatient unit. Three patients were admitted due to psychotic symptoms, 6 due a depression features and one due to manic symptoms. All of them were discharged to a minor intensity setting and none required of hospital admission after a month of the MH-HaH. At a year of follow-up, only one patient required a new hospital admission due to a relapse.

**Conclusions:**

MH-HaH programs could be a safe and respectful alternative to psychiatric admissions with a low relapse rate. However, the need of personalized approach of the dyad and the family as well as collaboration with the Perinatal Mental Health Units is required.

**Disclosure of Interest:**

None Declared

